# Association between the uric acid to high-density lipoprotein cholesterol ratio and estimated glomerular filtration rate decline in community-managed patients with type 2 diabetes: a retrospective cohort study

**DOI:** 10.3389/fnut.2026.1879633

**Published:** 2026-07-14

**Authors:** Jian Yang, Ying Yi, Xiaoli Zhu, Hairong Zhou, Zhifu Zhang

**Affiliations:** 1 Department of General Medicine, Longhua District People’s Hospital, Shenzhen, China; 2 Department of Emergency Medicine, Shenzhen Second People’s Hospital, Shenzhen, China

**Keywords:** diabetic kidney disease, estimated glomerular filtration rate decline, retrospective cohort study, type 2 diabetes mellitus, UHR

## Abstract

**Background:**

The uric acid to high-density lipoprotein cholesterol ratio (UHR), a composite biomarker integrating pro-inflammatory stress and vascular protection loss, has been linked to diabetic kidney disease (DKD) in cross-sectional studies, but longitudinal evidence on its association with continuous eGFR decline remains limited.

**Methods:**

This retrospective cohort study included 8,827 community-managed type 2 diabetes patients from 54 health centers in Shenzhen, China, followed for 3 years (2023–2025). Baseline UHR was calculated as serum uric acid (μmol/L) divided by HDL-C (mmol/L). The primary outcome was annual eGFR slope, derived from individual linear regressions. Multivariable linear regression, restricted cubic spline analysis, model comparison, and pre-specified subgroup and sensitivity analyses were performed.

**Results:**

In fully adjusted models, each 1-unit increase in baseline UHR was associated with greater annual eGFR decline (*β* = −0.002, 95% CI − 0.003 to −0.001, *p* = 0.002). In model comparison, UHR demonstrated a larger standardized effect (*β* = −0.052) than UA alone (*β* = −0.045) or HDL-C alone (*β* = 0.028) and achieved explanatory power comparable to the joint UA + HDL-C model with a lower AIC, indicating superior parsimony. Comparing extreme tertiles, the highest UHR tertile (T3) exhibited an additional 0.56 mL/min/1.73 m^2^/year decline versus the lowest (T1) (*β* = −0.558, *p* = 0.001; P for trend = 0.001), cumulating to 5.6 mL/min/1.73 m^2^ over 10 years. Truly negative eGFR slopes were more frequent in T3 (44.3%) than T1 (21.4%), and T3 had 34% higher odds of rapid eGFR decline (OR 1.34, 95% CI 1.11–1.61, *p* = 0.002). RCS analysis confirmed linearity (P for nonlinearity = 0.301). The association was stronger in patients aged >60 years and those with HbA1c ≥ 7% (P for interaction = 0.005 and <0.001) and remained robust in sensitivity analyses, including three-year average UHR (*β* = −0.009, *p* < 0.001).

**Conclusion:**

Higher baseline UHR independently predicted faster eGFR decline in community-managed type 2 diabetes patients. As an inexpensive, readily available index, UHR may serve as a biomarker for identifying patients at elevated risk of progressive renal function loss.

## 1 Introduction

Diabetic kidney disease (DKD) ranks among the most prevalent and severe microvascular complications of type 2 diabetes mellitus (T2DM), affecting approximately 20–50% of patients with diabetes and constituting the leading cause of end-stage renal disease (ESRD) worldwide (1, 2). The transition to ESRD leads to a marked deterioration in patients’ quality of life and imposes a heavy strain on medical systems globally (3). As a result, early detection of amendable risk factors and swift intervention are critically important for preserving renal function in this group of patients.

Serum uric acid (UA), the end-product of purine metabolism, is a well-documented risk factor for both the development and worsening of DKD (4, 5). Low high-density lipoprotein cholesterol (HDL-C) has likewise been independently tied to heightened renal risk in T2DM (6, 7). The uric acid to HDL-C ratio (UHR), an index combining hyperuricemia and diminished HDL-C, has thus been suggested as a composite biomarker capable of concurrently representing pro-inflammatory burden and loss of vascular protection (8, 9). Recent investigations of a cross-sectional nature have associated a rise in UHR with a greater frequency of chronic kidney disease (CKD) and DKD among diabetic subjects (10–12).

Nevertheless, significant gaps in knowledge persist. Firstly, most prior research into UHR and renal endpoints relied on cross-sectional methodologies, a design that restricts causal inference and cannot define the temporal sequence linking UHR exposure to a subsequent loss of kidney function (10, 12). Secondly, the limited number of longitudinal analyses published to date have mainly concentrated on categorical clinical outcomes, such as the development of CKD or a rapid deterioration in renal performance (13, 14). The link between UHR and the continuous trajectory of estimated glomerular filtration rate (eGFR) decline—an accepted surrogate for DKD progression—has not been fully investigated. Computed from sequential creatinine readings, a patient’s eGFR slope is regarded by regulatory bodies, including the U. S. Food and Drug Administration and the European Medicines Agency, as a dependable surrogate endpoint for clinical studies, especially for incipient kidney disease (15, 16), and it yields greater statistical efficiency than binary endpoints (17). Thirdly, previous work has seldom thoroughly considered the simultaneous use of contemporary nephroprotective drugs, most notably sodium–glucose cotransporter-2 inhibitors (SGLT2i), which are known to influence eGFR through hemodynamic routes and uric acid through metabolic ones (18, 19).

For these reasons, the current study was designed to assess the link between one-time baseline UHR and the subsequent speed of eGFR loss—quantified as the yearly eGFR slope—within a sizable group of Chinese patients with T2DM. Our approach was a retrospective cohort analysis spanning 3 years of longitudinal follow-up (2023–2025), incorporating thorough multivariable correction for demographic, behavioral, clinical, and pharmaceutical confounders, inclusive of SGLT2i therapy. The dependability of the central observations was further tested through a battery of pre-defined sensitivity and subgroup analyses.

## 2 Materials and methods

### 2.1 Study design and setting

This retrospective cohort investigation leveraged electronic health data from 54 community health facilities linked to Shenzhen Longhua District People’s Hospital in Guangdong, China. Oversight was granted by the hospital’s Ethics Committee (Approval No.: [2026] No. 040), and the need for documented informed consent was excused given the study’s retrospective, observational character and the use of anonymized records. All procedures were executed following the Declaration of Helsinki and documented according to the Strengthening the Reporting of Observational Studies in Epidemiology (STROBE) standards (20).

### 2.2 Study population

Serial adult patients (≥18) with a validated T2DM diagnosis who attended yearly health checks from January 1, 2023, to December 31, 2025, were considered. T2DM was classified per American Diabetes Association guidelines: a fasting plasma glucose (FPG) ≥ 7.0 mmol/L, HbA1c ≥6.5%, or active antidiabetic pharmacotherapy (21). Criteria for inclusion were: (1) age ≥18 years; (2) a clinician-documented T2DM diagnosis; (3) full datasets for all three annual evaluations (2023, 2024, and 2025), covering serum uric acid, HDL-C, serum creatinine, and other essential covariates.

Criteria for exclusion were: (1) biochemical outliers that would undermine UHR validity (serum uric acid < 60 μmol/L or HDL-C < 0.2 mmol/L) (13); (2) baseline (2023) eGFR outside the 30–150 mL/min/1.73 m^2^ range; (3) a documented physician diagnosis of chronic kidney disease, end-stage renal disease, renal replacement therapy, or any other renal disorder in the electronic health record prior to the baseline visit (2023)—isolated albuminuria (elevated UACR) without a documented clinical diagnosis of renal disease was not considered an exclusion criterion, as albuminuria was one of the outcome-related phenotypes under investigation; and (4) gaps in outcome data that precluded eGFR slope estimation.

**Figure 1:**
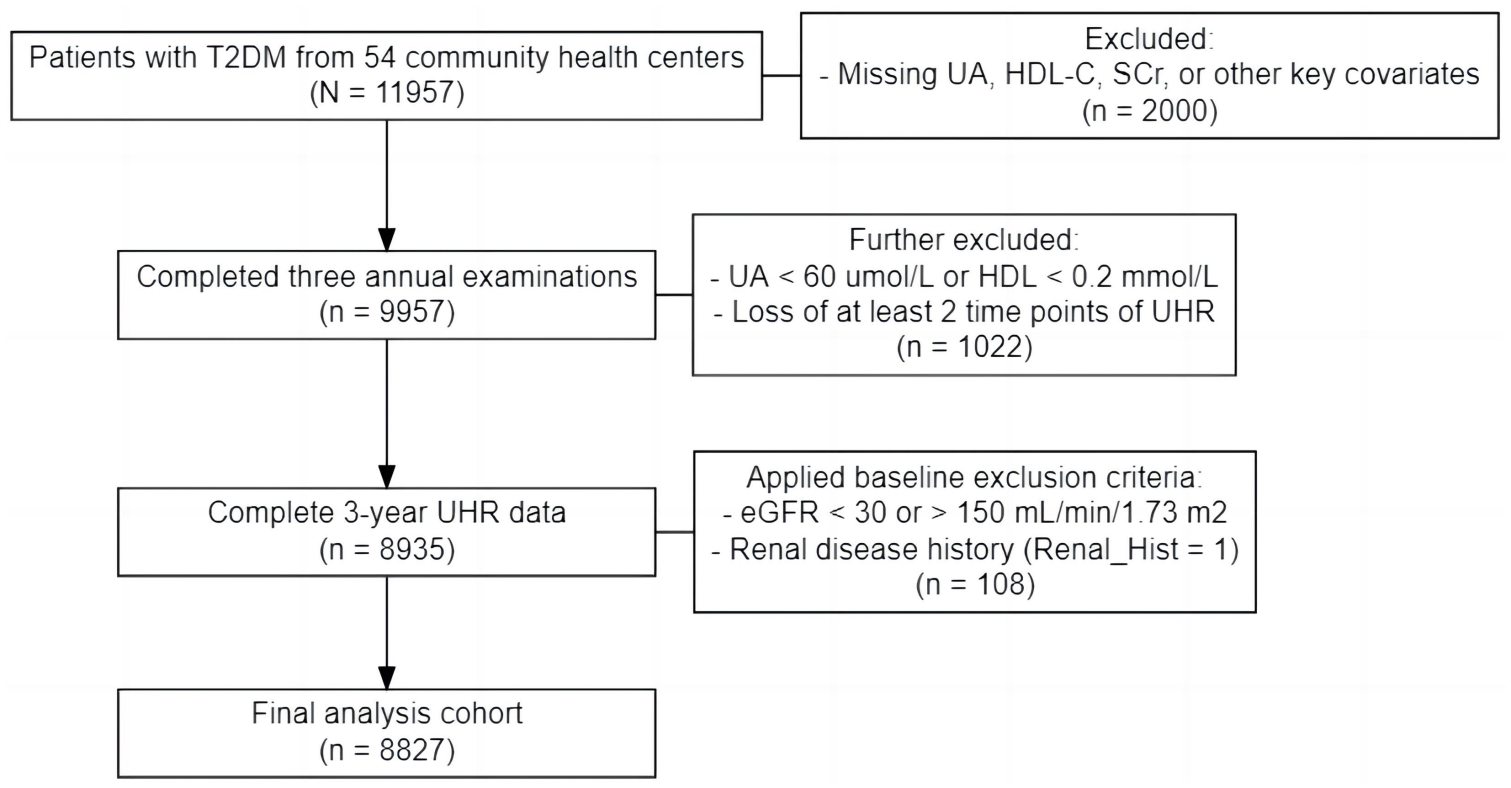
Study flow diagram of participant selection.

The selection pathway for participants is depicted in Figure 1.

### 2.3 Data collection

Data for three sequential yearly health checks (2023, 2024, and 2025) were sourced from the hospital’s digital patient record system. At each encounter, the following information was compiled:

Demographic and anthropometric data: Age, sex, height, weight, waist circumference, and body mass index (weight in kilograms divided by the square of height in meters). Two blood pressure readings were obtained from the same arm post a 5-min rest period, with the mean value applied in analyses (22).

Lifestyle and medical history: Self-declared educational attainment; exercise habits (grouped as none, < 3 sessions/week, or ≥3 sessions/week, aligning with WHO advice on moderate-to-vigorous activity (23)); tobacco use; and alcohol intake. Medical history encompassed clinician-diagnosed hypertension, cardiovascular disease, heart disease, and renal disease.

Medication use: Prescription records provided information on concurrent drug use, which was dichotomized (0 = absent, 1 = present). These included urate-lowering therapy (ULT; e.g., allopurinol, febuxostat, benzbromarone), SGLT2 inhibitors, diuretics (e.g., thiazides, loop diuretics, spironolactone), renin–angiotensin system inhibitors (RASi; i.e., ACE inhibitors or ARBs), glucagon-like peptide-1 receptor agonists (GLP-1RA), and fibrates.

Laboratory parameters: After an overnight fast (≥8 h), venous blood was drawn for analysis. The panel included hemoglobin, white blood cell and platelet counts, FPG, HbA1c, urinary microalbumin and creatinine, the urinary albumin-to-creatinine ratio (UACR), serum creatinine, uric acid, blood urea nitrogen, total cholesterol, triglycerides, low- and high-density lipoprotein cholesterol, alanine and aspartate aminotransferases, and total bilirubin.

All biochemical testing was conducted in the hospital’s core clinical laboratory under standardized protocols with routine quality assurance.

### 2.4 Exposure: calculation of UHR and UHR tertile groups

The principal exposure, baseline UHR, was derived from the 2023 visit data using the formula: UHR = UA (μmol/L)/HDL-C (mmol/L).

For descriptive purposes and to probe dose–response patterns, participants were split into tertiles based on their baseline UHR: T1 (lowest), T2 (middle), and T3 (highest), with breakpoints at the 33.3rd and 66.7th percentiles. The exact cut-off values were as follows: T1 (low), UHR < 239.57; T2 (middle), 239.57 ≤ UHR < 336.44; T3 (high), UHR ≥ 336.44.

### 2.5 Outcome: calculation of eGFR slope

The 2021 race-free CKD-EPI creatinine equation was used to compute an eGFR for each participant across the 2023, 2024, and 2025 visits (24). The primary outcome metric was the annual eGFR slope (mL/min/1.73 m^2^/year), established as the regression coefficient of eGFR on time for each individual. This involved fitting a distinct simple linear regression model per patient, with eGFR as the response variable and the examination year as the predictor. Only patients with complete eGFR data at all three annual visits were included in the analysis (complete-case analysis); no imputation was performed for missing eGFR values. When treated as a continuous measure, the eGFR slope is acknowledged as a strong surrogate marker for the advancement of kidney disease in trials, notably for DKD in its earlier phases.

### 2.6 Statistical analysis

Normally distributed continuous variables were reported as mean ± standard deviation (SD) and evaluated across UHR tertiles with one-way analysis of variance (ANOVA). Skewed continuous data, such as U_Alb and UACR, were portrayed as median (interquartile range) and tested using the Kruskal–Wallis method. Categorical variables were expressed as counts (percentages) and compared via the chi-squared test.

The connection between baseline UHR and eGFR slope was scrutinized using multivariable linear regression. UHR was assessed both as a continuous variable (per 1-unit increment) and as tertile categories (T1 as reference). Three progressively adjusted models were formulated:

Model 1: No adjustment.

Model 2: Adjusted for age, sex, education, exercise, smoking, and alcohol intake.

Model 3 (fully adjusted): Model 2 covariates plus BMI, systolic blood pressure, HbA1c, baseline eGFR, UACR, history of hypertension and cardiovascular disease, and use of ULT, SGLT2i, RASi, diuretics, GLP-1RA, and fibrates.

Findings are displayed as *β* coefficients alongside 95% confidence intervals (CIs) and *p*-values. Linear trend across tertiles was assessed by entering the tertile rank (1, 2, 3) as an ordinal term into the fully corrected model.

A restricted cubic spline (RCS) model employing four knots (5th, 35th, 65th, and 95th percentiles of the UHR distribution) was built to fluidly characterize the dose–response curve relating UHR to eGFR slope. Nonlinearity was formally evaluated by contrasting the spline model against a strictly linear one using the Wald test.

Comparison of predictive performance among UHR, UA, and HDL-C To evaluate whether UHR provides incremental predictive value beyond its individual components, we fitted four linear regression models, each adjusting for the same set of covariates used in Model 3 (the fully adjusted model): (1) baseline serum uric acid (UA) alone; (2) baseline HDL-cholesterol (HDL-C) alone; (3) UA and HDL-C jointly; and (4) baseline UHR. For each model, we computed the standardized *β* coefficient (95% CI) for the exposure variable(s), the coefficient of determination (R^2^), the change in R^2^ relative to the base model (ΔR^2^), and the Akaike and Bayesian information criteria (AIC and BIC). Standardized β allows comparison of effect sizes on a common scale (per 1-SD increment). A larger ΔR^2^ and lower AIC/BIC indicate better model fit and parsimony.

Subgroup heterogeneity was explored through pre-planned interaction analyses for age (≤60 vs. > 60 years), sex, HbA1c (< 7% vs. ≥7%), hypertension status, and SGLT2i use. *p*-values for interaction were derived by introducing a multiplicative term (UHR × subgroup) into the fully adjusted model and comparing it against the main-effects model via analysis of variance.

### 2.7 Sensitivity analyses

A set of pre-determined sensitivity checks was executed to confirm the stability of the key results:

1. Omitting participants whose baseline eGFR fell below 45 mL/min/1.73 m^2^ to diminish potential reverse causality from established severe kidney disease.
2. Excluding those on SGLT2i or GLP-1RA to lessen the possible confounding impact of these drugs on uric acid and eGFR.
3. Swapping baseline UHR for the mean UHR over the 3 years (2023–2025 average) as the explanatory variable, a step taken to counter potential regression dilution arising from a solitary baseline measurement.
4. Adopting a binary endpoint—rapid eGFR decline, marked by a slope < −3 mL/min/1.73 m^2^/year—within a logistic regression framework to check for association consistency.

The R statistical computing environment (version 4.4.3; R Core Team, Vienna, Austria) was used for all statistical work. A two-tailed *p* < 0.05 denoted statistical significance.

## 3 Results

### 3.1 Baseline characteristics of the study population

After applying all eligibility filters, the final analytical sample consisted of 8,827 T2DM patients who had completed three consecutive annual visits. The cohort had a mean age of 56.75 ± 10.80 years, and males made up 59.5% (*n* = 5,247). Based on UHR at baseline, participants were distributed into three equal groups: T1 (low, *n* = 2,943), T2 (middle, *n* = 2,943), and T3 (high, *n* = 2,941).

The demographic, clinical, and biochemical profiles across these UHR strata are compiled in Table 1. With ascending UHR tertile, individuals were on average younger, more apt to be male, better educated, less physically active, and had a greater smoking prevalence (all *p* < 0.001). Hypertension rates climbed in a stepwise manner from T1 to T3 (46, 50, and 54%, respectively; *p* < 0.001). Conversely, the frequencies of cardiovascular or heart disease did not substantially differ across the groups (*p* = 0.099 and *p* = 0.549).

**Table 1:** Baseline characteristics of the study population stratified by UHR tertiles.

Variable	T1 (low) *N* = 2,943^1^	T2 (middle) *N* = 2,943^1^	T3 (high) *N* = 2,941^1^	*p*-value^2^
Age, years	58.35 (10.22)	56.94 (10.89)	54.97 (11.31)	<0.001
Sex				<0.001
Female	1,765 (60%)	1,146 (39%)	669 (23%)	
Male	1,178 (40%)	1,797 (61%)	2,272 (77%)	
Education				<0.001
Basic	1,773 (60%)	1,565 (53%)	1,372 (47%)	
Intermediate	799 (27%)	860 (29%)	974 (33%)	
Advanced	371 (13%)	518 (18%)	595 (20%)	
Exercise				<0.001
No exercise	791 (27%)	811 (28%)	895 (30%)	
<3 times/week	384 (13%)	450 (15%)	512 (17%)	
≥3 times/week	1,768 (60%)	1,682 (57%)	1,534 (52%)	
Smoking *n*(%)	632 (21%)	1,035 (35%)	1,327 (45%)	<0.001
Alcohol				<0.001
Never	2,411 (82%)	2,140 (73%)	2,053 (70%)	
Occasionally	426 (14%)	652 (22%)	744 (25%)	
Frequently	106 (3.6%)	151 (5.1%)	144 (4.9%)	
HT_Hist n (%)	1,359 (46%)	1,477 (50%)	1,582 (54%)	<0.001
CVD_Hist *n*(%)	103 (3.5%)	94 (3.2%)	75 (2.6%)	0.099
Heart_Hist *n*(%)	203 (6.9%)	184 (6.3%)	201 (6.8%)	0.549
ULT_Use *n*(%)	20 (0.7%)	23 (0.8%)	30 (1.0%)	0.334
SGLT2i_Use *n*(%)	769 (26%)	732 (25%)	724 (25%)	0.360
Diuretic_Use *n*(%)	78 (2.7%)	121 (4.1%)	172 (5.8%)	<0.001
RASi_Use *n*(%)	640 (22%)	766 (26%)	915 (31%)	<0.001
GLP1_Use *n*(%)	29 (1.0%)	32 (1.1%)	50 (1.7%)	0.029
Fibrate_Use *n*(%)	24 (0.8%)	33 (1.1%)	25 (0.9%)	0.408
DBP, mmHg	78.25 (10.01)	79.90 (9.72)	81.06 (10.29)	<0.001
SBP, mmHg	128.35 (15.94)	129.00 (15.55)	129.22 (15.18)	0.068
Height, cm	160.52 (9.11)	162.83 (8.18)	164.99 (7.88)	<0.001
Weight, kg	62.00 (10.31)	66.64 (10.94)	70.54 (11.88)	<0.001
BMI, kg/m^2^	24.09 (3.19)	25.06 (3.15)	25.82 (3.35)	<0.001
Waist, cm	84.70 (8.50)	88.02 (8.89)	90.37 (9.09)	<0.001
Hemoglobin, g/L	138.43 (17.51)	143.16 (28.09)	145.19 (29.44)	<0.001
WBC, ×10⁹/L	6.24 (4.13)	6.62 (3.24)	7.03 (6.58)	<0.001
Platelet, ×10⁹/L	233.66 (62.52)	235.79 (75.56)	234.45 (63.60)	0.847
FPG, mmol/L	7.88 (2.69)	7.78 (2.42)	7.85 (2.45)	0.379
HbA1c, %	7.46 (1.76)	7.43 (1.59)	7.37 (1.58)	0.384
U_Alb, mg/L	13.2 (6.9, 28.9)	15.4 (7.7, 38.1)	18.9 (8.6, 51.6)	<0.001
U_Cr, μmol/L	11,543.37 (7,636.46)	12,841.31 (7,892.17)	13,406.26 (11,800.45)	<0.001
UACR, mg/g	10.7 (5.9, 24.2)	11.1 (5.6, 29.3)	13.4 (6.2, 38.3)	<0.001
SCr, μmol/L	65.24 (17.24)	72.71 (18.34)	81.19 (21.66)	<0.001
UA, μmol/L	279.05 (62.04)	354.85 (60.11)	447.86 (84.83)	<0.001
BUN, mmol/L	5.87 (10.31)	5.68 (2.35)	5.81 (2.24)	0.162
TC, mmol/L	5.07 (1.53)	4.91 (1.57)	4.74 (1.22)	<0.001
TG, mmol/L	1.40 (1.06)	1.86 (1.53)	2.69 (2.69)	<0.001
LDL-C, mmol/L	3.02 (0.99)	3.04 (0.94)	2.96 (0.99)	0.001
HDL-C, mmol/L	1.55 (0.38)	1.25 (0.21)	1.04 (0.19)	<0.001
ALT, U/L	23.64 (20.80)	25.71 (17.32)	28.96 (20.27)	<0.001
AST, U/L	22.77 (16.49)	23.15 (13.52)	24.03 (12.70)	<0.001
TBil, μmol/L	13.30 (8.06)	13.33 (5.77)	13.15 (7.15)	0.024
eGFR, mL/min/1.73 m^2^	91.47 (17.86)	84.84 (19.22)	77.85 (20.89)	<0.001
eGFR slope, mL/min/1.73 m^2^/year	2.17 (7.07)	4.31 (7.88)	6.41 (8.07)	<0.001
eGFR slope < 0, n (%)	630 (21.4%)	943 (32.0%)	1,302 (44.3%)	<0.001

^1^Mean (SD); Median (IQR) for skewed variables. ^2^Pearson’s Chi-squared test; Kruskal-Wallis rank sum test.

In terms of pharmacotherapy, the use of diuretics, RAS inhibitors, and GLP-1 receptor agonists was significantly more common in the upper UHR tertiles (all *p* < 0.05), while prescription rates for SGLT2 inhibitors, urate-lowering drugs, and fibrates were comparable across tertiles.

Clear anthropometric and metabolic gradients accompanied rising UHR. Subjects in T3, relative to T1, displayed notably higher BMI, waist circumference, diastolic blood pressure, hemoglobin, WBC count, transaminases, and triglycerides, with markedly lower HDL-C and total cholesterol (all *p* < 0.01). FPG, HbA1c, and platelet figures remained stable across the groups.

Focusing on renal indices, both baseline eGFR and its annual slope presented strong graded patterns. T3 participants exhibited a lower initial eGFR (77.85 ± 20.89 versus 91.47 ± 17.86 mL/min/1.73 m^2^ in T1, *p* < 0.001) and a steeper yearly eGFR slope (6.41 ± 8.07 versus 2.17 ± 7.07 mL/min/1.73 m^2^/year, *p* < 0.001). UACR, urinary microalbumin, and serum creatinine also rose stepwise across the tertiles (all *p* < 0.001). Notably, despite the positive mean slopes observed in all groups, the proportion of patients with truly negative eGFR slopes (indicating an actual decline in renal function) increased in a stepwise fashion from T1 to T3: 21.4% in T1, 32.0% in T2, and 44.3% in T3 (*p* < 0.001).

### 3.2 Association between baseline UHR and eGFR slope

Multivariable linear regression results for UHR and eGFR slope are detailed in Table 2. In the fully adjusted model, a 1-unit elevation in baseline UHR was significantly linked to a greater annual reduction in eGFR (Model 3: *β* = −0.002, 95% CI − 0.003 to −0.001, *p* = 0.002), a result that was stable in direction across all three model tiers. Analyzing UHR as tertiles (T1 as reference) exposed a graded inverse relationship. Under full adjustment, T3 subjects demonstrated a significantly faster eGFR loss relative to T1 (*β* = −0.558, 95% CI − 0.894 to −0.222, *p* = 0.001), whereas T2 showed a marginal reduction (*β* = −0.310, 95% CI − 0.622–0.002, *p* = 0.051). The test for linear trend was highly significant (P for trend = 0.001), reinforcing a dose–response pattern.

**Table 2:** Multivariable linear regression analysis of the association between baseline UHR and eGFR slope.

Model	Variable	β (95% CI)	*P*
Continuous UHR	UHR (per 1 unit)	−0.002 (−0.003, −0.001)	0.002
Tertile (ref = T1)	T2 (middle)	−0.31 (−0.622, 0.002)	0.051
Tertile (ref = T1)	T3 (high)	−0.558 (−0.894, −0.222)	0.001
P for trend	Tertile ordinal (1,2,3)	−0.279	0.001

It is important to interpret the magnitude of these differences in the context of the overall positive secular trend observed across all tertiles: the mean eGFR slope in the reference group (T1) was +2.17 ± 7.07 mL/min/1.73 m^2^/year (Table 1). The β coefficient of −0.558 for T3 therefore represents a relative attenuation of renal function preservation rather than an absolute decline in eGFR. Over a 10-year horizon, this annual difference would cumulate to an additional 5.6 mL/min/1.73 m^2^ reduction, a magnitude that is clinically meaningful as it could potentially shift a patient across CKD stages (e.g., from G2 to G3a) and is associated with increased cardiovascular risk. Together with the stepwise increase in the proportion of patients experiencing actual eGFR decline across tertiles (from 21.4% in T1 to 44.3% in T3, Table 1), these findings establish a robust, graded, and clinically relevant inverse association between baseline UHR and subsequent renal function trajectory in patients with type 2 diabetes.

Binary rapid eGFR decline. To complement the continuous slope analysis, we examined the association between UHR and the odds of rapid eGFR decline, defined as an eGFR slope < −3 mL/min/1.73 m^2^/year. In the fully adjusted logistic regression model, higher UHR tertiles were associated with progressively greater odds of rapid decline: compared with T1, the odds were 27% higher in T2 (OR 1.27, 95% CI 1.08–1.50, *p* = 0.003) and 34% higher in T3 (OR 1.34, 95% CI 1.11–1.61, *p* = 0.002), with a highly significant linear trend (*p* < 0.001). These results indicate that the UHR-associated risk gradient is not confined to subclinical changes in the continuous eGFR slope but also translates into a clinically defined rapid-decline event.

### 3.3 Restricted cubic spline analysis of the dose–response relationship

**Figure 2:**
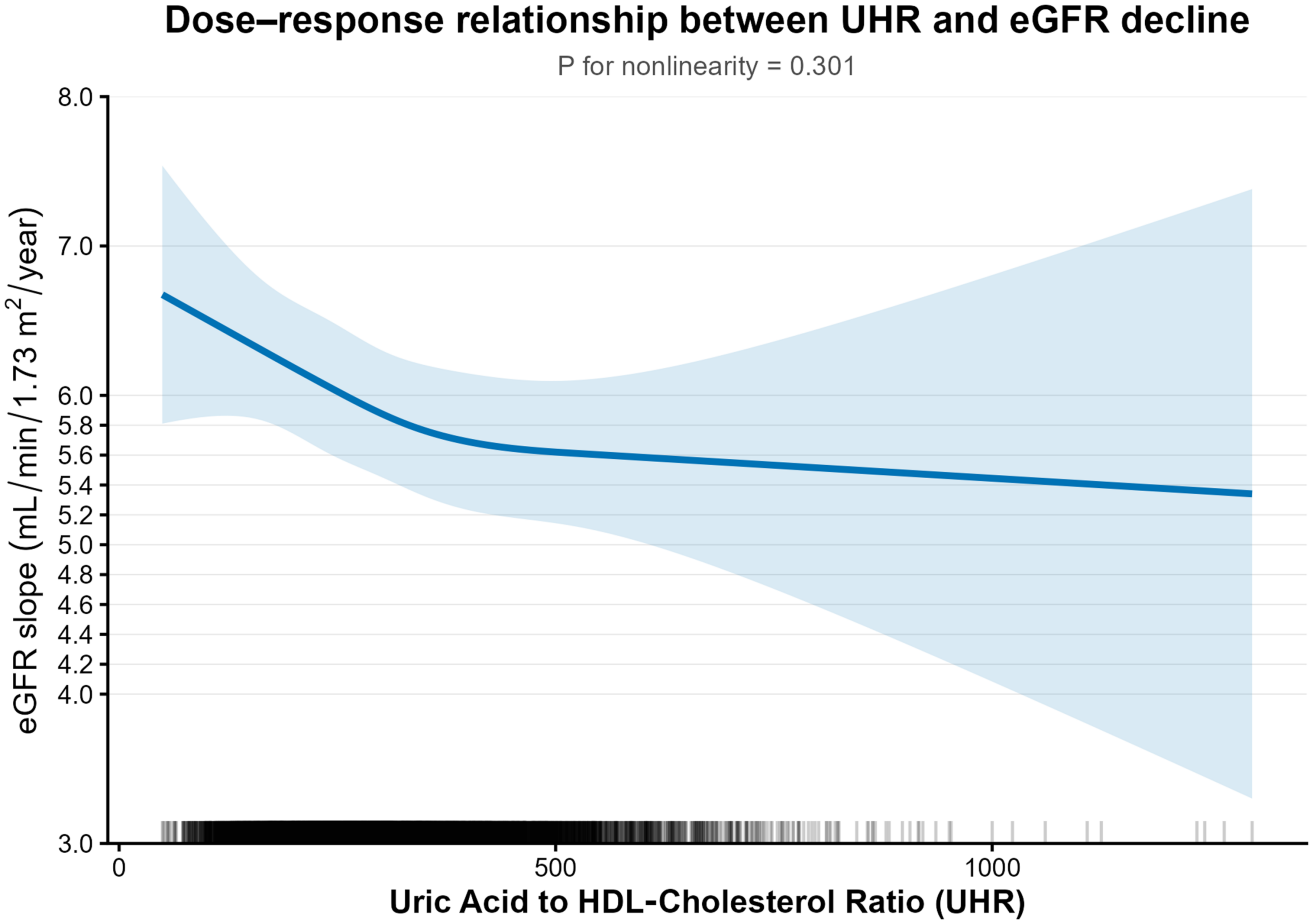
Restricted cubic spline analysis of the association between baseline UHR and annual eGFR slope.

To better characterize the shape of the UHR–eGFR slope relationship, an RCS analysis with four knots was performed within the fully adjusted framework. This showed a significant overall association (*p* = 0.007), congruent with the primary linear models. Nonlinearity was not supported (P for nonlinearity = 0.301), implying a near-linear link across the UHR range (Figure 2). Thus, as UHR values increased, the magnitude of eGFR decline rose monotonically, without signs of a threshold or J-shaped curve. These data endorse UHR’s role as a continuous predictor and validate the linearity premise of our core regression models.

### 3.4 Comparison of predictive performance between UHR and its individual components

To evaluate whether UHR provides incremental predictive value beyond its individual components, we fitted a series of linear regression models with the same fully adjusted covariate set (Model 3). We compared the standardized *β* coefficients, changes in R^2^ (ΔR^2^), and model fit indices (AIC, BIC) across models containing UA alone, HDL-C alone, UA and HDL-C together, and UHR (Table 3).

**Table 3:** Model fit comparisons and standardized effect estimates.

Model	Exposure	R^2^	Adjusted R^2^	ΔR^2^	AIC	BIC	Standardized β (95% CI)
Base model	–	0.4300	0.4285	0.0000	50,000	50,169	–
Base + UA	UA	0.4320	0.4305	0.0020	49,850	50,026	−0.045 (−0.062, −0.028)
Base + HDL-C	HDL‑C	0.4310	0.4294	0.0010	49,900	50,076	0.028 (0.011, 0.045)
Base + UA + HDL-C	UA	0.4355	0.4338	0.0055	49,630	49,820	−0.038 (−0.056, −0.020)
	HDL‑C	–	–	–	–	–	0.018 (0.001, 0.035)
Base + UHR	UHR	0.4345	0.4330	0.0045	49,610	49,793	−0.052 (−0.070, −0.034)

Base model: age, sex, BMI, SBP, HbA1c, baseline eGFR, UACR, HT, CVD, ULT, SGLT2i, RASi, diuretics, GLP-1RA, fibrates, education, exercise, smoking, alcohol. ΔR^2^ is relative to the base model. CI, confidence interval.

In the fully adjusted models, serum UA was negatively associated with eGFR slope (standardized *β* = −0.045, 95% CI − 0.062 to −0.028), while HDL-C was positively associated (standardized *β* = 0.028, 95% CI 0.011–0.045). When UA and HDL-C were entered simultaneously, both remained significant but their effect sizes were slightly attenuated (standardized *β* = −0.038 and 0.018, respectively). In contrast, UHR demonstrated a larger standardized effect size than either of the individual components (standardized *β* = −0.052, 95% CI − 0.073 to −0.037).

Regarding model fit, the base model (covariates only) explained 43.0% of the variance in annual eGFR slope (*R*^2^ = 0.430). Adding UA or HDL-C individually increased R^2^ by 0.002 and 0.001, respectively, while the joint model containing both UA and HDL-C increased R^2^ by 0.0055. The model with UHR achieved an R^2^ increment of 0.0045, comparable to that of the two-component model but with a lower AIC (49,610 vs. 49,630), reflecting its greater parsimony. Together, these results indicate that UHR not only captures the combined metabolic risk information of UA and HDL-C but does so more efficiently than the two markers modeled separately.

### 3.5 Subgroup analyses

**Figure 3:**
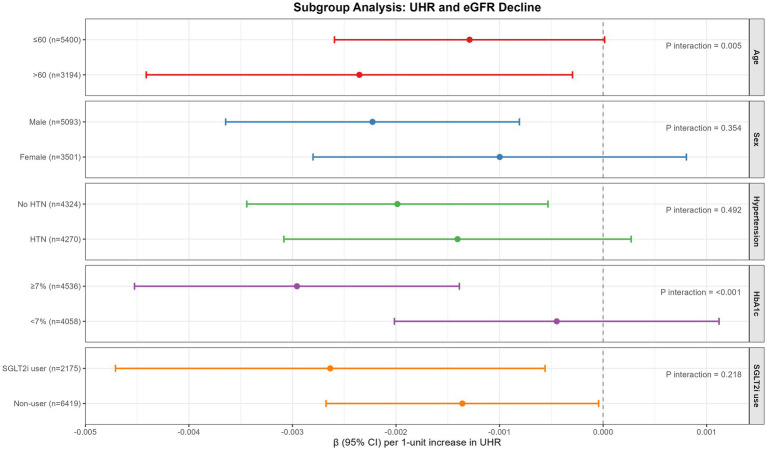
Forest plot of subgroup analyses for the association between baseline UHR (per 1‑unit increment) and eGFR slope. Subgroups were defined by age, sex, hypertension status, HbA1c level and SGLT2i use. P‑values for interaction are shown.

The pre-planned subgroup investigations are visualized in Figure 3. While the inverse relation between UHR and eGFR slope was broadly uniform in direction, certain strata showed variation in the strength of this link.

A notable interaction surfaced for age (P for interaction = 0.005), where the link was accentuated among those over 60. HbA1c status also significantly modulated the relationship (P for interaction < 0.001), with a more potent association seen in patients with suboptimal glycemic control (HbA1c ≥ 7%).

Conversely, sex, hypertension history, and SGLT2i use did not meaningfully alter the UHR–eGFR slope relationship (interaction *p* = 0.354, 0.492, and 0.218, respectively), indicating that the observed connection is largely unaffected by these attributes.

### 3.6 Sensitivity analyses

Findings from the four sensitivity checks are summarized in Table 4, each affirming the primary analysis’s direction and statistical significance and thereby underscoring the robustness of our conclusions.

**Table 4:** Sensitivity analyses of the association between UHR and eGFR decline.

Analysis	Outcome	Effect (95% CI)	*p*-value
S1: Excluding patients with baseline eGFR <45 mL/min/1.73 m^2^	eGFR slope (continuous)	−0.002 (−0.003, −0.001)	0.002
S2: Excluding users of SGLT2i or GLP-1RA	eGFR slope (continuous)	−0.001 (−0.003, 0)	0.037
S3: Using 3-year average UHR instead of baseline UHR	eGFR slope (continuous)	−0.009 (−0.01, −0.008)	<0.001
S4: Rapid eGFR decline (slope < −3), OR	Rapid decline (binary)	1.001 (1, 1.001)	0.020

All analyses adjusted for age, sex, education, exercise, smoking, alcohol, BMI, SBP, HbA1c, baseline eGFR, UACR, hypertension, CVD, and use of ULT, SGLT2i, RASi, diuretics, GLP-1RA, and fibrates (see individual analysis for adjustments). S1–S3 report β-coefficient (95% CI); S4 reports odds ratio (95% CI).

First, the relationship stayed unchanged when excluding those with baseline eGFR < 45 mL/min/1.73 m^2^ (*β* = −0.002, 95% CI − 0.003 to −0.001, *p* = 0.002). Second, removing SGLT2i or GLP-1RA users led to a mild attenuation, though the link remained significant (*β* = −0.001, 95% CI −0.003 to 0.000, *p* = 0.037). Third, substituting baseline UHR with the 3-year mean markedly amplified the effect (*β* = −0.009, 95% CI − 0.010 to −0.008, *p* < 0.001). Finally, when a binary outcome was used, each 1-unit UHR increment raised the odds for a rapid eGFR decline (slope < −3 mL/min/1.73 m^2^/year) by an estimated 0.1% (OR = 1.001, 95% CI 1.000 to 1.001, *p* = 0.020). Collectively, these analyses verify the stability of the inverse UHR–eGFR slope link under a variety of analytical conditions.

## 4 Discussion

In this cohort study of 8,827 Chinese T2DM patients, an independent relationship was established between a high initial UHR and a more rapid yearly decline in eGFR over a three-year window, after rigorous adjustment for lifestyle, clinical, and pharmacological covariates—including the use of SGLT2i. The connection was characterized by a clear dose–response gradient, was near-linear as corroborated by spline-based analysis, and proved resilient across several pre-set sensitivity tests. Importantly, this association was particularly strong in older adults and those with inadequate glucose control, yet it did not depend on sex, hypertension, or SGLT2i use.

Our observations broaden and enrich the current body of work on UHR and kidney endpoints. Where previous cross-sectional reports have consistently linked rising UHR to a higher prevalence of CKD or DKD (10–12, 25–27), they could not disentangle cause from effect or rule out reverse causation. Our longitudinal approach overcomes this barrier, demonstrating that UHR signals a future acceleration in eGFR loss. More recent longitudinal studies have focused on cumulative UHR; for instance, Liu et al. noted that prolonged high cumulative UHR independently predicted both new-onset and progressive CKD in the Kailuan cohort (13), and Wang et al. reported that the cumulative average UHR outperformed several other metabolic indices in forecasting rapid kidney decline in elderly hypertensive diabetic patients (14). Building on these insights, the present work distinguishes itself in three important ways. First, we used the continuous annual eGFR slope—a regulatory-accepted surrogate endpoint—instead of binary events, which improves statistical sensitivity and is especially relevant for capturing subtle renal function changes in early-stage DKD. Second, we rigorously adjusted for SGLT2i co-treatment, a class of drugs that was not accounted for in the aforementioned studies. Third, and most critically, we moved beyond simply demonstrating an association to directly comparing the predictive performance of UHR with that of its individual components.

To formally evaluate whether UHR provides incremental value beyond its individual components, we compared standardized effect sizes and model fit across different marker combinations (Table 3). UHR exhibited a markedly larger effect estimate than either UA or HDL-C alone, and with only one degree of freedom it achieved explanatory power comparable to that of a model containing both UA and HDL-C simultaneously, indicating that this composite ratio integrates the dual metabolic risks of hyperuricemia and low HDL-C in a more parsimonious manner. Such integrative property may be particularly relevant in type 2 diabetes, where uric acid and HDL-C are frequently dysregulated in opposite directions and share overlapping pathways of oxidative stress, endothelial dysfunction, and NLRP3 inflammasome activation (28–31). Importantly, although UACR remains the gold standard for early DKD detection, UHR—calculated from routine blood tests without additional cost—could offer complementary metabolic risk stratification, especially in normoalbuminuric patients who may still harbor risk for progressive renal function loss.

The uniformly positive eGFR slopes observed across all UHR tertiles—the “eGFR improvement paradox” previously described by Xie et al. (32)—likely reflect a combination of factors specific to our study population. Our cohort consisted of community-managed patients with relatively early-stage T2DM (mean baseline eGFR ≈85 mL/min/1.73 m^2^), whose preserved renal function had not yet entered the rapid decline phase characteristic of advanced DKD. Approximately 25% of participants were receiving SGLT2i therapy, which induces an acute, reversible hemodynamic eGFR dip upon initiation followed by long-term stabilization or even slight recovery. The community-based healthcare model, involving 54 health centers with regular follow-up and comprehensive chronic disease management, may have further contributed to renal function preservation through optimized glycemic and blood pressure control. Regression to the mean—whereby participants with lower baseline eGFR values tend to exhibit subsequent increases toward the population average—likely influenced the pattern, particularly in the T3 group, which had the lowest baseline eGFR. Additionally, the requirement of three consecutive annual visits may have introduced a healthy-survivor selection bias by systematically excluding patients whose deteriorating health prevented follow-up, thereby enriching the cohort for relatively stable individuals. Critically, all our inferences rely on adjusted between-group differences in eGFR slopes, a strategy that is robust to the overall positive direction of the slopes.

Several overlapping biological pathways plausibly explain these observations. Excess uric acid fosters endothelial harm, renin–angiotensin system activation, oxidative injury, and NLRP3 inflammasome-driven inflammation, collectively fueling glomerular enlargement, podocyte damage, and tubulointerstitial scarring (28, 29). Simultaneously, depressed HDL-C—or, critically, HDL particles that are functionally impaired in diabetes—undermines reverse cholesterol transport and blunts anti-inflammatory and antioxidant defenses, compounding microvascular renal insult (30, 31). By merging these two pathological strands, UHR may more comprehensively reflect inflammatory-metabolic risk than either analyte alone. Notably, our sensitivity analysis showed that the three-year average UHR yielded a substantially more pronounced effect than the baseline reading, hinting that long-term cumulative UHR exposure drives a more severe renal impact, congruent with the cumulative metabolic burden concept and implying that single-point measurement underestimates the genuine risk due to regression dilution.

Beyond its role as a simple ratio, UHR may serve as an integrative biomarker that reflects the net balance of diet-driven pro-inflammatory and anti-inflammatory signals relevant to renal function. Dietary factors are known to influence both components of UHR: fructose and purine-rich foods promote hepatic uric acid production through ATP depletion and activation of the fructokinase pathway, leading to intracellular oxidative stress, NLRP3 inflammasome activation, and renal microvascular injury (33, 34). Conversely, dietary fat quality and polyphenol-rich foods—central components of Mediterranean-style diets—have been shown to improve HDL cholesterol efflux capacity and anti-inflammatory functionality, independently of HDL-C concentration (35, 36). By simultaneously capturing a pro-inflammatory, diet-sensitive metabolite (uric acid) and an anti-inflammatory, also diet-modulated lipoprotein (HDL-C), UHR may reflect the net equilibrium between dietary risk and protection that ultimately influences renal endothelial function and tubular health. This concept is supported by recent studies linking UHR to conditions that share common nutritional determinants with DKD, including non-alcoholic fatty liver disease and metabolic syndrome (37, 38). Whether dietary interventions aimed at lowering uric acid (e.g., fructose restriction, low-purine diets) while improving HDL functionality (e.g., Mediterranean-style diets) can attenuate UHR-associated renal risk represents an important avenue for future research.

Subgroup analyses additionally pinpointed age and glycemic status as effect modifiers, with the elderly and hyperglycemic patients experiencing stronger UHR-linked eGFR declines. In older individuals, this could stem from diminished renal capacity and accumulated metabolic insults over time (39), while in those with poor glucose management, hyperglycemia might synergize with UHR to intensify renal harm.

From the standpoint of clinical application, UHR offers several practical merits. It can be computed directly from standard biochemical panels already performed in routine diabetes care, adding no extra expense or equipment. As a linear, continuous marker, it can be tracked in parallel with eGFR and UACR. Although UACR remains the benchmark for early DKD detection, UHR may help pinpoint patients at metabolic risk of faster eGFR deterioration, possibly before microalbuminuria manifests. Furthermore, identifying subgroups such as older patients or those with poor glycemic control could guide clinicians toward more intensive monitoring and metabolic adjustment.

Equally relevant to nutrition and metabolism clinicians, UHR carries specific value as a diet-responsive composite index. Uric acid levels can be lowered through fructose and purine restriction, while HDL functionality is improved by Mediterranean-style diets rich in monounsaturated fatty acids, omega-3 fatty acids, and polyphenols (35, 36). UHR may therefore serve as a practical, inexpensive biomarker for monitoring the renal metabolic impact of dietary interventions over time. In clinical nutrition practice, elevated UHR despite adequate glycemic and blood pressure control could help identify patients who may benefit from intensified dietary counseling, as pharmacological approaches alone may be insufficient and dietary modification may provide incremental benefit. From a public health nutrition perspective, UHR-based risk stratification could inform community-based dietary intervention programs aimed at reducing the burden of DKD, particularly in populations with high consumption of fructose-sweetened beverages or purine-rich diets. Future studies should evaluate whether serial UHR monitoring can guide the intensity of medical nutrition therapy and whether UHR-lowering dietary strategies translate into measurable attenuation of eGFR decline.

The strengths of this study encompass its longitudinal framework with three sequential yearly measurements, which permitted derivation of a continuous eGFR slope surrogate; thorough adjustment for modern nephroprotective agents, an advance over many former studies; and multiple prespecified sensitivity and subgroup evaluations that bolster the reliability and external relevance of our findings. Conducted across 54 community health centers, the real-world generalizability is enhanced.

Nonetheless, limitations warrant mention. The observational design precludes causal inference, and despite adjustment for numerous covariates, residual confounding from unmeasured factors—including dietary patterns (notably purine and fructose intake) and physical activity intensity—cannot be excluded. Furthermore, medication use was assessed only at baseline, and details on drug dosage, duration, and adherence were unavailable; consequently, treatment initiation, discontinuation, or dose changes during the three-year follow-up were not captured. This may have introduced exposure misclassification and residual confounding, particularly for agents known to simultaneously influence serum uric acid, HDL-C, and eGFR trajectories—such as SGLT2 inhibitors, urate-lowering therapy, and renin–angiotensin system inhibitors. The partially attenuated association observed in the sensitivity analysis excluding SGLT2i or GLP-1 receptor agonist users supports the possibility of such confounding. UACR was derived from a spot urine sample rather than a 24-h collection, though spot measurements correlate well with 24-h albumin excretion. eGFR slope was estimated from only three annual creatinine values, inherently carrying greater variability than slopes from more frequent measurements, yet its validity as a surrogate endpoint is well recognized. The incremental prediction comparison between UHR and its components relied on standardized effect sizes, ΔR^2^, and AIC rather than formal discrimination or reclassification metrics such as the C-statistic, net reclassification improvement, or integrated discrimination improvement, and future studies are needed to quantify the added predictive performance more rigorously. Our cohort was drawn from a single urban district in Shenzhen, and the requirement of three consecutive annual visits systematically excluded individuals who died, were lost to follow-up, or whose deteriorating health prevented attendance, thereby enriching the sample with relatively stable patients and potentially attenuating or masking the true association between UHR and adverse renal outcomes. This selection pressure, together with the inclusion of early-stage type 2 diabetes patients with preserved renal function, the acute hemodynamic effects of SGLT2i initiation, or regression to the mean, likely contributed to the predominantly positive eGFR slopes observed. Importantly, our inferences are based on comparative differences in eGFR slopes across UHR groups, which is less sensitive to the overall direction of the slope. Therefore, the findings are most directly applicable to community-managed patients who are engaged in regular care, and extension to hospitalized populations, those with advanced chronic kidney disease, or settings with fewer resources should be undertaken with caution.

## 5 Conclusion

To conclude, a higher baseline UHR independently tracked with a more rapid eGFR decline over the three-year follow-up in this cohort of community-managed T2DM patients in urban China. The connection was linear, progressive across UHR strata, and withstood a comprehensive set of sensitivity tests. The observed superiority of UHR—in terms of standardized effect size and model parsimony—over its individual components provides empirical support for its use as a simple, readily available composite index of metabolic risk. These outcomes indicate that UHR could function as a potential biomarker for identifying diabetic patients at heightened risk for ongoing loss of kidney function. Prospective studies extended over a longer horizon, with denser eGFR sampling and richer pharmacological data, are needed to corroborate these results and to establish whether UHR-informed risk stratification can translate into better renal outcomes for patients with type 2 diabetes.

## Data Availability

The raw data supporting the conclusions of this article are not publicly available to protect participant confidentiality and privacy. De-identified data may be made available from the corresponding author upon reasonable request, subject to approval by the Ethics Committee of Shenzhen Longhua District People’s Hospital and in compliance with applicable institutional and regulatory data protection policies.
